# Impaired but now diagnosable: Combining analytic frameworks for incorporating symptom weighting in the classification of full‐threshold and subthreshold posttraumatic stress disorder

**DOI:** 10.1002/jts.70112

**Published:** 2026-08-05

**Authors:** Krithika Prakash, Antonio A. Morgan‐López, Jordan A. Gette, Kelly R. Peck, Marilyn L. Piccirillo, Lissette M. Saavedra, Sudie E. Back, Therese K. Killeen, Sonya B. Norman, Stephen G West, Denise A. Hien, the Consortium on Addiction, Stress, and Trauma

**Affiliations:** ^1^ Department of Psychiatry University of Pittsburgh Pittsburgh Pennsylvania USA; ^2^ RTI International Research Triangle Park North Carolina USA; ^3^ Department of Psychiatry and Behavioral Sciences University of Kansas Medical Center Kansas City Kansas USA; ^4^ Department of Psychiatry University of Vermont Burlington Vermont USA; ^5^ Department of Psychiatry Rutgers Robert Wood Johnson Medical School New Brunswick New Jersey USA; ^6^ Rutgers Addiction Research Center Brain Health Institute Rutgers Health Piscataway New Jersey USA; ^7^ Department of Psychiatry and Behavioral Sciences Medical University of South Carolina Charleston South Carolina USA; ^8^ Ralph H. Johnson VA Healthcare System Charleston South Carolina USA; ^9^ National Center for PTSD–White River Junction White River Junction Vermont USA; ^10^ Department of Psychiatry University of California–San Diego La Jolla California USA; ^11^ Department of Psychology Arizona State University Tempe Arizona USA; ^12^ Center of Alcohol and Substance Use Studies Rutgers University–New Brunswick New Brunswick New Jersey USA; ^13^ Department of Clinical Psychology Rutgers University–New Brunswick New Brunswick New Jersey USA

## Abstract

Individuals with subthreshold posttraumatic stress disorder (PTSD) often experience significant impairment yet face barriers to care (e.g., insurance denials and exclusion from clinical trials) due to strict *Diagnostic and Statistical Manual of Mental Disorders* (*DSM*)–based diagnostic criteria that rely entirely on symptom counts. Symptom counts assume all symptoms contribute equally to underlying (within‐criterion) severity. Recently, item response theory and nonlinear factor analysis models have been used to estimate PTSD severity, accounting for differences in the relative weight of symptoms. We examined a subset of treatment‐seeking adults with comorbid PTSD and substance use disorders in Project Harmony who met the criteria for full PTSD based on weighted severity scores regardless of whether they met the full *DSM‐*defined criteria. Latent class analysis was then used to identify differences in symptom endorsement patterns in this empirically estimated full‐threshold PTSD group. Three distinct classes were identified: (a) participants with full‐threshold PTSD based on both *DSM* and weighted classification, (b) underdiagnosed participants who did not endorse either *DSM* Criterion C (avoidance) symptom, or (c) underdiagnosed participants who only endorsed one *DSM* Criterion E (alterations in arousal/reactivity) symptom. Endorsement of two *DSM* Criterion D symptoms (negative alterations in cognitions and mood) did not differ across classes, suggesting that they could be “priority symptoms” indicating high distress and impairment, regardless of *DSM* diagnostic status or severity level. Accounting for symptom weighting and prioritizing these symptoms in assessment and diagnosis could increase precision in PTSD assessment and ensure equitable access to care.

Trauma exposure is a pervasive experience; recent data show that approximately 70% of people globally have experienced at least one traumatic event in their lifetime (Kessler et al., 2017). However, lifetime prevalence of posttraumatic stress disorder (PTSD) is around 6.1%, indicating that most individuals with trauma exposure do not go on to develop the disorder (Schein et al., 2021). Among individuals who develop PTSD, presentations are highly heterogeneous (Galatzer‐Levy & Bryant, 2013). The fifth edition of the *Diagnostic and Statistical Manual of Mental Disorders* (*DSM‐5*; American Psychiatric Association [APA], 2013) and subsequent text revision (*DSM‐5*‐*TR*; APA, 2022) note that the diagnostic criteria for full‐threshold PTSD requires meeting Criterion A (i.e., exposure to actual or threatened death, serious injury, or sexual violence), along with meeting at least one of five intrusion symptoms in Criterion B, one of two avoidance symptoms in Criterion C, two of seven negative alterations in cognitions and mood symptoms (NACM) in Criterion D, and two of six alterations in arousal and reactivity (AAR) symptoms in Criterion E.

A standardized definition of PTSD is useful for treatment planning, referrals, and reimbursement. However, many individuals may not meet the criteria for a PTSD diagnosis following exposure to a Criterion A event despite experiencing significant distress and impairment (Hoge et al., 2014; Kilpatrick et al., 2013). Two potential issues may help explain why these individuals may be underdiagnosed: (a) the lack of consensus in how subthreshold PTSD is defined and (b) the assumption in the *DSM* framework that all symptoms contribute equally to underlying symptom severity.

## Definitions and operationalization of subthreshold PTSD

PTSD severity that is related to clinical distress but does not meet the *DSM* criteria is referred to as *subthreshold PTSD*. Subthreshold PTSD is associated with a number of physical and mental health conditions that can significantly impact quality of life and functioning (Carty et al., 2006; El‐Gabalawy et al., 2018; Marshall et al., 2001). Individuals with subthreshold PTSD are less able to access PTSD‐specific treatment and are often excluded from clinical trials that rely on a PTSD diagnosis for inclusion (Cukor et al., 2010; Dickstein et al., 2013; Kornfield et al., 2012; Norman et al., 2007).

We posit that one reason this population is overlooked is due to the lack of consensus in defining subthreshold PTSD. Clinical researchers have proposed a range of definitions for subthreshold PTSD, from conservative approaches (e.g., the omission of a single symptom from a single criterion; Franklin et al., 2018) to more liberal approaches (e.g., requiring only one symptom per criterion; Carlier & Gersons, 1995; Stein et al., 1997). However, these definitions have primarily been developed based on clinical judgement rather than empirical evidence. Although some studies, such as those by Franklin et al. (2018) and Mylle and Maes (2004), have retrospectively analyzed how the application of these definitions affects diagnostic classifications, the definitions themselves have not been formulated through empirical methods. Additionally, although the *DSM‐5* and *DSM‐5‐TR* currently include the “other specified trauma‐ and stressor‐related disorder” diagnosis (APA, 2013, 2022) to capture individuals who do not meet the criteria for full‐threshold PTSD, this designation is arguably insufficient, as it still does not account for the differential weight of specific symptoms.

## The problem of equal symptom weighting in PTSD diagnosis

A de facto assumption of the current *DSM* framework is that individuals who meet the full‐threshold diagnostic criteria for PTSD inherently experience greater distress and impairment than those who experience subthreshold symptoms. However, this assumption has been challenged both theoretically (Galatzer‐Levy & Bryant, 2013) and empirically (Morgan‐López et al., 2024). Research using statistical methods such as nonlinear factor analysis (NLFA) and item response theory (IRT) has found that a nontrivial proportion of individuals with subthreshold PTSD experience higher symptom severity than some patients with a full‐threshold *DSM*‐based diagnosis (i.e., higher latent PTSD scores with fewer symptoms endorsed; Morgan‐López, Killeen et al., 2020; Morgan‐López, Saavedra et al., 2020). These models are designed to account for differences in the relative strength of the association between the latent variable (i.e., PTSD severity) and the probability of endorsement for each symptom. Discrimination parameters in IRT or factor loadings in NLFA can be thought of as “clinical weights” representing the severity of each symptom.

Multiple studies have consistently demonstrated that some PTSD symptoms have higher psychometric weight than others for effectively distinguishing between severity levels (Contractor et al., 2018; Morgan‐López, Killeen, et al., 2020; Ruglass et al., 2020; Saavedra et al., 2021, 2022; Silverstein et al., 2020). By relying on an “equal weights” model, *DSM*‐based diagnosis may underestimate clinical severity and result in poorer outcomes, as well as a limited understanding of the disorder's impact on individuals (impairment and distress) and society (prevalence rates, economic and public health burden; Cukor et al., 2010; Marshall et al., 2001).

To address these limitations of an equal‐weights model, Morgan‐López, Killeen, et al. (2020) proposed an approach to PTSD diagnosis using moderated nonlinear factor analysis (MNLFA; Bauer, 2017). This approach accounts for measurement noninvariance by modeling differences in the relative weights and prevalences of PTSD symptoms at equivalent levels of latent PTSD severity. The first step in the MNLFA approach is to weight PTSD symptoms based on their factor loadings to estimate a latent weighted PTSD severity score. The second step is to estimate an empirical grouping threshold (EGT) by computing the midpoint between the mean PTSD severity scores of individuals with a full‐threshold PTSD diagnosis and those with subthreshold PTSD, adjusted for their respective standard deviations (see Jacobson and Truax's [1991] normative threshold formula):

$$Empirical\;Cut-off=\frac{\mu_{clinical}\sigma_{normative}+\mu_{normative}\sigma_{clinical}}{\sigma_{normative}+\sigma_{clinical}}$$

Here, *normative* refers to patients with no diagnosis (i.e., subthreshold PTSD), whereas *clinical* refers to patients with a full‐threshold PTSD diagnosis. This is consistent with Jacobson and Truax's (1991) recommendation that “an agreed upon community standard” (e.g., diagnostic groups) be used to distinguish the clinical and normative distributions of a scale score. Participants who fell below this cutoff on the latent weighted PTSD score were grouped in the subthreshold weighted PTSD classification group, and those who were above the cutoff were grouped in the full‐threshold weighted PTSD classification. These weighted classifications were compared against full‐threshold and subthreshold *DSM* PTSD diagnoses, creating a 2x2 table of four groups characterized by individuals with: (a) a full‐threshold *DSM* PTSD diagnosis and a full‐threshold weighted PTSD classification, (b) a subthreshold *DSM* PTSD diagnosis and subthreshold weighted PTSD classification, (c) a full‐threshold *DSM* PTSD diagnosis but a subthreshold weighted PTSD classification, and (d) a subthreshold *DSM* PTSD diagnosis but a full‐threshold weighted PTSD classification.

Using this approach in Morgan‐López, Killeen, et al. (2020), participants with a full‐threshold *DSM* PTSD diagnosis who also had a full‐threshold weighted PTSD classification had higher symptom endorsement rates on 15 of 17 PTSD symptoms, as outlined in the fourth edition of the *DSM* (*DSM‐IV*; APA, 1994), compared to those with a full‐threshold *DSM* PTSD diagnosis but a subthreshold weighted PTSD classification. A sensitivity analysis showed a larger separation of PTSD treatment outcome trajectories between participants with full‐threshold and subthreshold weighted PTSD classifications (*d* = 0.99) compared to trajectory differences for full‐threshold versus subthreshold *DSM* diagnoses (*d* = 0.24; Morgan‐López et al., 2023), further solidifying the clinical utility and predictive validity of distinguishing full‐threshold and subthreshold PTSD based on relative weights of PTSD symptom levels. These findings provide strong evidence that the *DSM* classification obscures meaningful differences in underlying severity.

## Current study objectives

These prior findings clarified the symptom configurations of individuals with full‐threshold *DSM‐*defined PTSD and weighted subthreshold PTSD. However, it remains unclear which specific symptom patterns most commonly characterize individuals with a subthreshold *DSM* PTSD diagnosis but a full‐threshold weighted PTSD classification. The current analysis was based on a subgroup of treatment‐seeking individuals presenting with comorbid PTSD and substance use disorders (SUDs) in Project Harmony (Morgan‐López et al., 2022). These individuals had a full‐threshold weighted PTSD classification regardless of *DSM*‐based diagnoses, and they also met the full *DSM* criteria for PTSD (i.e., concordant) or did not (i.e., underdiagnosed). This current article presents a latent class analysis (LCA; Muthén & Muthén, 1998–2017) using partial training data of PTSD symptoms from harmonized clinical interviews in this subset of participants. The goal was to identify classes of symptom patterning that included mixtures of (a) an observed class of participants who also had a full‐threshold *DSM* PTSD diagnosis and (b) latent classes of participants who did not meet the criteria for a full‐threshold *DSM* PTSD diagnosis (i.e., underdiagnosed) among the remainder of the subsample. After class enumeration, differences in the symptom endorsement probabilities across concordant versus underdiagnosed classes were examined to see which symptoms were related to *DSM*‐based underdiagnosis and which symptoms did not differ across classes. The results provide initial empirical support for specific symptom patterns that prioritize PTSD severity based on weighted symptoms rather than the simple symptom counts (within‐criterion). The goal of this research is to help reduce the likelihood of PTSD underdiagnosis.

## METHOD

### Participants and procedure

The sample included a subset (*n* = 1,568) of participants enrolled in one of 25 randomized clinical trials of individuals with comorbid PTSD and SUDs who completed semistructured PTSD interviews (*N* = 2,658). Data from 25 clinical trials were included in Project Harmony, an individual‐participant meta‐analysis designed to examine the efficacy of behavioral and/or pharmacological interventions for comorbid PTSD and substance use disorders (Morgan‐López et al., 2022). To select the current subsample, we applied a symptom‐weighting classification strategy (Morgan‐López, Killeen, et al., 2020; Morgan‐López et al., 2023). Specifically, weighted PTSD symptom severity was estimated using MNLFA, and participants were selected if their weighted severity score crossed the EGT required for full‐threshold weighted classification, regardless of *DSM* diagnostic status.

Most participants identified as White (*n* = 1,021, 65.1%), followed by Black (*n* = 389, 24.8%), Hispanic (*n* = 111, 7.1%), Asian (*n* = 14, 0.9%), or “other” (*n* = 66, 4.2%). The average participant age was 39.36 years (*SD* = 11.44), and 659 (42.0%) participants identified as male. Participants reported having a high school education or less (*n* = 541, 34.5%), having completed some college (*n* = 382, 24.3%), being a college graduate (*n* = 194, 12.4%), or they did not report their level of educational attainment (*n* = 451, 28.8%). There were 560 participants (35.7%) who identified as veterans.

### Measures

Baseline measures from Project Harmony datasets were analyzed. Several clinician‐administered interviews were used to assess *DSM‐IV* and *DSM‐5* PTSD symptoms: the Clinician‐Administered PTSD Scale (CAPS; Blake et al., 1995; *k* = 21); the CAPS for *DSM‐5* (CAPS‐5; Weathers et al., 2013; *k* = 2); and the PTSD Symptom Scale–Interview (PSS‐I; Foa et al., 1993; *k* = 2). The CAPS‐IV assessed the frequency and intensity of 17 *DSM‐IV* PTSD symptoms over 30 days to determine PTSD diagnostic status and symptom severity and demonstrated high internal consistency, Cronbach's α = .94, and test–retest reliability, *r*s = .90–.98 (Blake et al., 1995). The PSS‐I assessed distress caused by the 17 *DSM‐IV* PTSD symptoms in the past month and demonstrated strong internal consistency, Cronbach's α = .89, and test–retest reliability, *r* = .87 (Foa et al., 1993). The CAPS‐5 measured the frequency and intensity of the 20 *DSM‐5* PTSD symptoms in the past 30 days and has shown high internal consistency, Cronbach's α = .88, and good test–retest reliability, intraclass correlation coefficient (ICC) = .78 (Weathers et al., 2013, 2018). Using item harmonization (Bauer & Hussong, 2009), PTSD items were standardized into a binary (0/1) format, resulting in a total of 21 symptoms, used as latent class indicators, based on the published item‐to‐symptom conversion approach for each assessment system. These included 16 common symptoms across both *DSM‐IV* and *DSM‐5*, and five noncommon symptoms (i.e., foreshortened future from *DSM‐IV* only, and negative beliefs, distorted blame, persistent negative emotional state, and reckless/self‐destructive behavior from *DSM‐5*). Details regarding specific data cleaning and harmonization procedures can be found in Morgan‐López et al. (2022).

### Data analysis

The purpose of the current analysis was to identify common patterns of PTSD symptoms that meet the criteria for weighted full‐threshold PTSD but may or may not meet the full‐threshold *DSM* diagnostic criteria. The primary analysis focused on an LCA of the full‐threshold weighted PTSD subset and examined differences, or lack thereof, in symptom endorsement rates between classes in this subset of participants. Latent class models were fit to the harmonized PTSD symptoms among the 1,568 participants identified in Morgan‐López et al., 2023 who met the full‐threshold weighted PTSD criteria. All data analyses were conducted using M*plus* (Version 8; Muthén & Muthén, 1998–2017). An iterative process was used to estimate the number of latent classes; the best‐fitting models were determined using combinations of the Akaike information criterion (AIC) and Bayesian information criterion (BIC). With training data, the likelihood ratio test (LRT) and the bootstrap LRT are not available for M*plus* mixture modeling. When indices disagreed, the BIC was prioritized, as it has shown the highest accuracy in recovering the correct number of classes across simulation studies (Morgan, 2015; Nylund‐Gibson & Choi, 2018). Entropy and average posterior probabilities were examined as indicators of classification quality.

Once the final latent‐class structure was identified, we characterized specific symptom patterns by calculating the endorsement probability of each symptom across classes. We conducted a series of binary logistic regressions to determine the likelihood of symptom endorsement in a one‐step model in M*plus* (Muthén, 2001). This approach avoids the bias associated with treating latent classes as manifest variables and ensures that classification uncertainty (i.e., posterior probabilities) is incorporated into the parameter estimates.

Finally, to delineate which symptoms should be prioritized for making diagnoses, we descriptively reviewed the specific *DSM* criterion that precluded a diagnosis (e.g., failing to meet Criterion C vs. Criterion D vs. Criterion E) among underdiagnosed individuals. We grouped underdiagnosed individuals by comparing symptom endorsement rates across latent classes. The magnitude of symptom differences between groups was quantified via Cohen's *h* (Cohen, 1988), where an *h* value greater than |1.0| indicated a large meaningful difference in symptom presentations across groups, and an *h* value less than |0.5| indicated nonmeaningful differences. Symptoms that did not differ between the underdiagnosed groups relative to the concordant group were considered to be potential “priority” symptoms regardless of *DSM* diagnostic status.

## RESULTS

### LCA results

Results of the LCA indicated a three‐class solution showed the best fit to the data based on AIC and BIC metrics. Although a four‐class solution yielded the lowest AIC (26,387 vs. 26,462 for the three‐class model), the three‐class model yielded the lowest BIC (26,808 vs. 26,850 for the four‐class model) and was thus selected as the final model. This solution resulted in an entropy of .99 and high posterior probabilities (range: .93–1.00), suggesting good distinction between classes (see Figure 1 and Supplementary Table S1).

**FIGURE 1:**
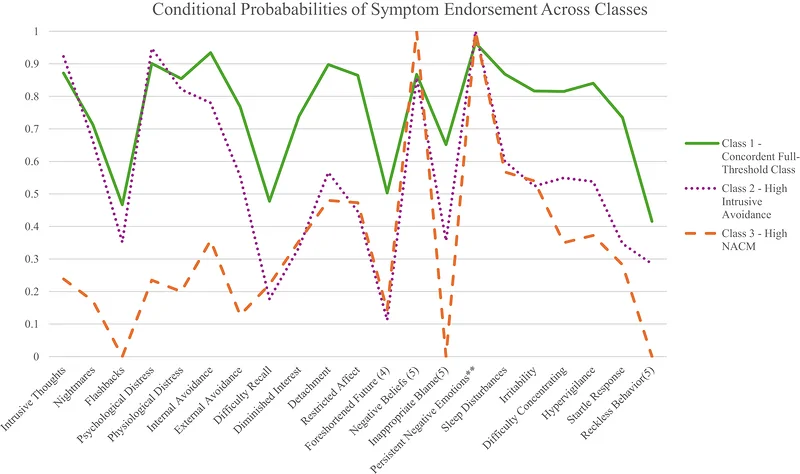
Conditional probabilities of symptom endorsement across classes. *Note*: The “(4)” designation indicates that the symptom is included only in the fourth edition of the *Diagnostic and Statistical Manual of Mental Disorders* (*DSM‐IV*). The “(5)” designation indicates that the symptom is included only in the fifth edition of the *DSM* (*DSM‐5*). NACM = negative alterations in cognitions and mood.

The largest group, Class 1 (*n* = 1,373, 90.7%), termed the *concordant full‐threshold class* (i.e., *diagnostic class*), was characterized by high endorsement probabilities across all criteria and was composed of individuals who met both the *DSM* and weighted classifications for full‐threshold PTSD. The second class (Class 2) was characterized by higher endorsement of intrusion (Criterion B) and avoidance (Criterion C) symptoms and lower endorsement of NACM (Criterion D) symptoms and was termed the *high intrusive–avoidance* class (*n* = 69, 4.6%). Participants in the third class (Class 3) demonstrated lower‐severity intrusion (Criterion B) and avoidance (Criterion C) symptoms and higher NACM (Criterion D) symptoms, and this class was termed the *high NACM* class (*n* = 72, 4.76%).

Both Class 2 and Class 3 were composed of individuals who met the full‐threshold weighted PTSD classification but not the full‐threshold *DSM* criteria (i.e., underdiagnosed; *n* = 141). Although these two classes each comprised slightly less than 5% of the sample, the large overall sample size (*N = *1,514) and high entropy (.989) indicate that these classes were well‐separated and distinct and support the validity of the three‐class solution despite the lower prevalence of the subtypes. Further, (a) the base rate for underdiagnosed participants would not be expected to be large and (b) this smaller subset was found to be replicable in an independent sample (Morgan‐López, Killeen et al., 2020).

### Symptom endorsement probabilities and effect sizes across classes

Next, symptom endorsement probabilities were compared across classes to aid in interpreting the relevant symptom‐level differences that help define the classes (see Table 1). Participants in the concordant full‐threshold class demonstrated higher odds of endorsing most symptoms, odds ratios (*OR*s) = 1.26–7.99, except intrusive thoughts, psychological distress related to trauma reminders, negative cognitions, inappropriate blame, persistent negative emotions (e.g., shame or guilt), and reckless behavior, when compared to those in the high intrusive–avoidance class. Similarly, participants in the concordant full–threshold class demonstrated higher odds of endorsing nearly all symptoms, *OR*s = 3.21–29.74, except negative cognitions and beliefs and persistent negative emotions, when compared to those in the high NACM class.

**TABLE 1:** Odds ratios (*OR*s) of posttraumatic stress disorder (PTSD) symptom indicators among latent classes.

	Class 1 vs. Class 2		Class 1 vs. Class 3		Class 2 vs Class 3	
PTSD symptom	*OR*	95% CI	*OR*	95% CI	*OR*	95% CI
*DSM‐5* Criterion B: Intrusions						
Intrusive thoughts	0.567	[0.140, 2.305]	21.635^*^	[9.110, 51.384]	38.125	[7.528, 193.079]
Nightmares	1.264^*^	[0.607, 2.630]	12.026^*^	[5.201, 27.806]	9.515	[2.849, 31.777]
Flashbacks	1.613^*^	[0.852, 3.055]	‐^a^		‐^a^	
Psychological distress	0.503	[0.097, 2.617]	29.743^*^	[10.998, 80.439]	59.099	[8.284, 421.600]
Physiological distress	1.279^*^	[0.502, 3.264]	23.487^*^	[11.167, 49.399]	18.357	[5.941, 56.719]
*DSM‐5* Criterion C: Avoidance						
Internal avoidance	4.017^*^	[1.898, 8.502]	25.607^*^	[13.668, 47.974]	6.374^*^	[2.418, 16.803]
External avoidance	2.663^*^	[1.489, 4.763]	22.705^*^	[8.413, 61.280]	8.525	[2.500, 29.066]
*DSM‐5* Criterion D: Negative alterations in cognitions and mood						
Difficulty recall	4.236^*^	[2.127, 8.436]	3.210^*^	[1.730, 5.959]	0.758^*^	[0.282, 2.039]
Diminished interest	5.600^*^	[3.007, 10.428]	5.148^*^	[2.868, 9.240]	0.919^*^	[0.354, 2.387]
Detachment	6.722^*^	[3.653, 12.368]	9.515^*^	[5.386, 16.810]	1.416	[0.577, 3.476]
Restricted affect	7.951^*^	[4.377, 14.444]	7.134^*^	[4.111, 12.380]	0.897^*^	[0.372, 2.161]
Foreshortened future^d^	7.985*	[2.991, 21.315]	6.104^*^	[2.776, 13.420]	0.764	[0.188, 3.103]
Negative beliefs^e^	1.085	[0.221, 5.338]	0.000	[0.000, 0.000]	0.000	[0.000, 0.000]
Inappropriate blame^e^	3.384	[1.062, 10.781]	Infinite^*^		Infinite^*^	
Persistent negative emotions^e^	0.000	[0.000, 0.000]	0.000	[0.000, 0.000]	1.000	[1.000, 1.000]
*DSM*‐*5* Criterion E: Alterations in arousal and reactivity						
Sleep disturbances	4.392^*^	[2.227, 8.662]	5.023^*^	[2.772, 9.105]	1.144	[0.417, 3.136]
Irritability	4.032^*^	[2.149, 7.565]	3.782^*^	[2.073, 6.901]	0.938	[0.346, 2.544]
Difficulty concentrating	3.610^*^	[1.909, 6.826]	8.184^*^	[4.517, 14.828]	2.267^*^	[0.892, 5.763]
Hypervigilance	4.519^*^	[2.581, 7.913]	8.869^*^	[4.998, 15.740]	1.963^*^	[0.836, 4.606]
Startle response	5.205^*^	[2.828, 9.581]	7.045^*^	[3.863, 12.850]	1.354^*^	[0.534, 3.430]
Reckless behavior^e^	1.787	[0.529, 6.038]	Infinite^*^		Infinite^*^	

*Note*. Class 1 = *Concordant full‐threshold class* standard with participants who met both *Diagnostic and Statistical Manual of Mental Disorders* (*DSM*)– and model‐based classifications for full PTSD; Class 2 = high intrusive and avoidance symptoms; Class 3 = high negative alterations in cognitions and mood symptoms. *DSM‐5* = *DSM* (5th ed).

^a^ Odds ratio is inestimable because the conditional proportion within Class 3 is 0.0.

^b^ Odds ratio is inestimable because the conditional proportion within Class 3 is 1.0

^c^ Indicates an undefined or extremely high odds ratio due to a near‐zero base rate in the comparison group.

^d^ Symptom included only in the fourth edition of the *DSM* (*DSM‐IV*).

^e^ Symptom included only in the *DSM‐5*.

* *p* < .05.

### Examinations of symptom patterns among underdiagnosed participants

We observed three patterns that occurred relatively frequently among *DSM*‐subthreshold participants: (a) participants who met Criteria B, D, and E but failed to meet Criterion C (*n* = 20 [14.2%] of underdiagnosed participants), (b) participants who met Criteria B, C, and E but failed to meet Criterion D (*n* = 18 [12.8%] of underdiagnosed participants), and (c) participants who met Criteria B, C, and D but failed to meet Criterion E (*n* = 36 [25.5%] of underdiagnosed participants).

#### Underdiagnosed participants missing Criterion C (avoidance)

Participants who failed to meet Criterion C (i.e., avoidance) had an average PTSD MNLFA severity score of 0.58 standard deviations above the cutoff score used for model‐based classification of full‐threshold PTSD and were only 0.25 standard deviations lower than the average severity score for participants in the concordant full‐threshold class (0.44 vs. 0.69). Participants who did not meet Criterion C were significantly less likely than those in the concordant full‐threshold class to endorse the following symptoms: internal avoidance of trauma‐related thoughts, external avoidance of trauma‐related cues, diminished interest, foreshortened future, and inappropriate blame, *h*s > 1.0. Physiological distress, restricted affect, negative beliefs, persistent negative emotions, sleep disturbances, and concentration problems were endorsed with lower frequency among participants in this subgroup compared with those in the concordant full‐threshold group. These differences did not represent medium or large effect sizes, *h*s < 0.5 (Table 2). Interestingly, irritability was more common in this subgroup compared to the concordant full‐threshold subgroup. The symptom profile that reflected the minimum number of symptoms across Criteria B–D for this group included one intrusion symptom, no avoidance symptoms, three or more NACM symptoms (most commonly negative beliefs and persistent negative emotions), and at least three AAR symptoms (most commonly sleep disturbances and irritability).

**TABLE 2:** Symptom pattern endorsement rates and effect sizes across underdiagnosed versus concordant full‐threshold (full‐threshold) class participants.

PTSD symptom	Full‐threshold (%)	MoA (%)	Full‐threshold vs. MoA (effect size*)	Mo NACM (%)	Full‐threshold vs. Mo NACM (effect size*)	Mo AAR (%)	Full‐threshold vs. Mo AAR (effect size)
*DSM‐5* Criterion B: Intrusions							
Intrusive thoughts	87.1	65.0	0.53	66.7	0.50	80.6	0.18
Nightmares	71.3	30.0	0.85	66.7	0.10	55.6	0.33
Flashbacks	46.7	15.0	0.71	27.8	0.39	27.9	0.39
Psychological distress	90.1	60.0	0.73	78.1	0.33	69.4	0.53
Physiological distress	85.5	70.0	0.38	66.7	0.45	58.3	0.62
*DSM‐5* Criterion C: Avoidance							
Internal avoidance	93.4	0.0	2.62 ^d^	83.3	0.32	91.7	0.07
External avoidance	76.8	0.0	2.14^d^	61.1	0.34	61.2	0.34
*DSM‐5* Criterion D: NACM							
Difficulty recall	47.8	15.4	0.72	0.0	1.53^d^	30.6	0.35
Diminished interest	73.9	25.0	1.02^d^	0.0	2.07^d^	66.7	0.16
Detachment	89.8	55.0	0.82	0.0	2.49^d^	86.1	0.11
Restricted affect	86.5	70.0	0.41	0.1	2.32^d^	61.1	0.59
Foreshortened future^b^	50.3	0.1	1.51^d^	5.6	1.10^d^	16.8	0.73
Negative beliefs^c^	86.7	79.6	0.19	77.6	0.24	79.5	0.19
Inappropriate blame^c^	65.2	5.7	1.40^d^	6.9	1.35^d^	10.7	1.21^d^
Persistent negative emotions^c^	96.5	89.1	0.29	87.0	0.36	91.0	0.23
*DSM*‐*5* Criterion E: AAR							
Sleep disturbances	86.8	70.0	0.42	72.2	0.37	16.9	1.55^d^
Irritability	81.6	100.0	0.89	77.8	0.10	27.8	1.15^d^
Difficulty concentrating	81.5	70.0	0.27	55.7	0.57	17.1	1.40^d^
Hypervigilance	84.0	45.0	0.85	66.7	0.41	16.7	1.48^d^
Startle response	73.5	45.0	0.59	61.6	0.26	0.0	2.06^d^
Reckless behavior^c^	41.6	17.1	0.55	13.6	0.65	9.8	0.76

*Note*. Effect sizes were calculated as Cohen's *h*. PTSD = posttraumatic stress disorder; *DSM‐5* = *Diagnostic and Statistical Manual of Mental Disorders* (5th ed.); MoA = miss on avoidance (participants who met Criteria B, D, and E but not Criterion C); MoNACM = miss on negative alterations in cognitions and mood (participants who met Criteria B, C, and E but not Criterion D); MoAAR = miss on alterations in arousal/reactivity (participants who met Criteria B, C, and D but not Criterion E).

^a^ Includes participants who met both *DSM‐*defined and model‐based classifications for full PTSD.

^b^ Symptom included only in the fourth edition of the *DSM* (*DSM‐IV*).

^c^ Symptom included only in the *DSM‐5*.

^d^ Cohen's *h* > |1.0|

#### Underdiagnosed participants missing Criterion D (NACM)

Participants who failed to meet Criterion D (i.e., NACM) had an average PTSD MNLFA severity score 0.75 standard deviations above the cutoff score used for model‐based classification of full‐threshold PTSD and were only 0.08 standard deviations lower than the average severity score for concordant full‐threshold participants (0.61 vs. 0.69). Participants who did not meet Criterion D were significantly less likely than those in the concordant full‐threshold class to endorse the following symptoms: difficulty in recalling the trauma, diminished interest, detachment, restricted affect, foreshortened future, and inappropriate blame, *h*s > 1.0; however, they showed no differences from concordant full‐threshold participants on negative beliefs, persistent negative emotions, thought avoidance, activity avoidance, sleep disturbances, or irritability, *h*s < 0.5. The symptom profile that reflected the minimum number of symptoms across Criteria B–D for this subgroup included two or more intrusion symptoms, one avoidance symptom, and at least two AAR symptoms (most commonly sleep disturbances and irritability).

#### Underdiagnosed participants missing Criterion E (AAR)

Participants who failed to meet Criterion E (i.e., AAR) had an average PTSD MNLFA severity score 0.88 standard deviations above the cutoff score used for model‐based classification of full‐threshold PTSD and were only 0.05 standard deviations lower than the average severity score for concordant full‐threshold participants (0.74 vs. 0.69). Participants who did not meet Criterion E were significantly less likely than concordant full‐threshold participants to endorse the following symptoms: inappropriate blame, sleep disturbances, irritability, difficulty concentrating, hypervigilance, and exaggerated startle response, *h*s > 1.0. Participants in this subgroup showed no meaningful differences compared to concordant full‐threshold participants regarding the endorsement of intrusive thoughts, nightmares, flashbacks, internal avoidance, external avoidance, difficulty recalling the trauma, diminished interest, detachment, negative beliefs, and persistent negative emotions, *h*s < 0.5. The symptom profile that reflected the minimum number of symptoms across Criteria B–D for this subgroup included one intrusion symptom (most commonly intrusive thoughts), one avoidance symptom (no particular pattern of symptom priority), two negative alterations in cognition and mood symptoms (most commonly negative beliefs and shame/guilt), and one AAR symptom (no particular pattern of symptom priority).

## DISCUSSION

Psychological responses to trauma exposure vary (Galatzer‐Levy & Bryant, 2013), and the current *DSM* criteria do not adequately predict clinically meaningful distress and impairment. Accordingly, many trauma‐exposed individuals do not receive a diagnosis and are limited in access to care. Symptom‐weighting approaches to diagnosing PTSD may improve assessments of distress and impairment. This study investigated symptom profiles and endorsement patterns among individuals who did not meet the *DSM* diagnostic criteria for PTSD but reported high levels of PTSD symptom severity under an empirical, symptom‐weighted model.

Results from the LCA demonstrated three distinct classes of individuals, each with unique symptom profiles. Although almost 91% of participants met both the *DSM* and weighted criteria for PTSD, slightly less than 10% of individuals did not meet the *DSM* criteria but exhibited underlying PTSD symptom severity equivalent to or exceeding the severity for those with full‐threshold diagnoses. This discrepancy highlights a substantial diagnostic gap, as many of these individuals narrowly missed diagnostic thresholds, often by just one criterion or even a single symptom. Specifically, individuals in the high intrusive–avoidance class experienced considerable trauma‐related impairment yet failed to meet the *DSM* criteria primarily due to insufficient endorsement of NACM symptoms (Criterion D). Participants in the high negative cognition class demonstrated notable distress but did not meet the diagnostic criteria due to relatively lower endorsement of intrusion and avoidance symptoms.

Despite these class‐level differences, two specific symptoms showed no variation in endorsement rates across the classes: persistent negative beliefs (e.g., “I am broken,” “no one can be trusted,” “the world is dangerous”) or negative feelings (e.g., fear, horror, shame, guilt). It is important to acknowledge that many symptoms are not specific to PTSD and share significant overlap with depression and anxiety; thus, these elevations could potentially be driven by comorbid conditions rather than PTSD alone. Regardless of whether these symptoms represent only PTSD or comorbid distress, our results indicate they consistently mark high levels of impairment across all classes, even when other diagnostic criteria (e.g., avoidance) are absent. We refer to these as potential “priority” symptoms for PTSD. It is also noted that in psychometric studies of the CAPS‐5, negative cognitions and persistent negative feelings are often among the symptoms with the highest item information—that is, these symptoms are good at differentiating between people with low versus high levels of underlying PTSD (Lee et al., 2024; Silverstein et al., 2020).

Clinically, this finding implies that individuals who experience persistent negative beliefs or emotions demonstrate levels of clinical severity consistent with levels exhibited by those with a PTSD diagnosis, even if they do not fully meet the *DSM* criteria for avoidance or AAR symptoms. However, clinicians should also consider other co‐occurring conditions (e.g., major depression or anxiety; APA, 2022).

This study underscores a broader challenge in PTSD research. The lack of formal recognition for these individuals can lead to the persistence of two systemic barriers. First, restrictive inclusion criteria in clinical research limit access to evidence‐based care for this population. Many randomized clinical trials (RCTs) for PTSD interventions exclude individuals who do not meet the full‐threshold *DSM* diagnostic criteria, despite evidence that subthreshold PTSD symptoms are clinically meaningful (Cukor et al., 2010; Dickstein et al., 2013; Kornfield et al., 2012). To our knowledge, most samples do not include individuals with subthreshold PTSD, with few exceptions (e.g., Hien et al., 2015; Ronconi et al., 2014; Ruglass et al., 2017). Thus, there is limited research to determine whether empirically supported PTSD treatments are similarly effective for individuals with subthreshold PTSD. This issue is particularly concerning for underserved populations that already face barriers in accessing treatment (Davis et al., 2008; Ouimette et al., 2011). By relying on strict categorical cutoffs during screening, there is a risk of systematically excluding these groups from studies, thereby limiting their access to one of the few affordable treatment options and introducing bias into and limiting the generalizability of research.

Second, strict *DSM*‐based diagnostic restrictions directly impact access to treatment in clinical settings. Some integrated health care settings, such as Veterans Affairs (VA) hospitals in the United States, may allow patients to access care without meeting strict PTSD diagnostic criteria. However, for many private and nonresearch clinical settings, insurance providers are often restrictive, typically requiring a *DSM*‐defined diagnosis to reimburse treatment costs (Lazar et al., 2018). Aligning the diagnostic criteria with demonstrated distress and impairment would expand coverage for many individuals, offering a more affordable avenue to access care. Until such changes occur, clinicians will remain without the tools or support to fully meet their clients’ needs, and many individuals who experience significant distress will lack access to treatment.

The primary limitation of this study involves the specific makeup of the sample. Project Harmony was originally designed as a meta‐analysis of individual patient data regarding treatment for comorbid PTSD and SUDs (Morgan‐López et al., 2022). Given that different studies used different ways to measure outcomes (e.g., CAPS, CAPS‐5, PSS‐I), we used integrative data analysis methodologies for item‐level harmonization (Curran et al., 2008). Our sample represents treatment‐seeking comorbid PTSD/SUD patients; the majority of these trials had a *DSM*‐based PTSD diagnosis as one of the inclusion criteria. Only a handful of trials also allowed patients with subthreshold PTSD into the trials (e.g., Hien et al., 2015; Ruglass et al., 2017). We also included data from individuals who were screened but did not qualify for the final trials because they did not meet the criteria for full‐threshold PTSD. Because our sample consisted entirely of individuals who were actively seeking treatment for co‐occurring substance use problems, the specific symptom profiles observed here must be interpreted within this clinical context. Additionally, the cross‐sectional nature of these baseline PTSD assessments fails to account for symptom fluctuations across one's life. Thus, these underdiagnosed individuals may be in a stage of onset or remission when specific symptoms have either not yet manifested or have already resolved. For instance, in our treatment‐seeking sample, participants who did not meet Criterion C (avoidance) may have intentionally reduced avoidance as part of their ongoing or prior treatment.

However, no single sample is uniquely suited to answer questions regarding psychiatric classification and nosology. Although other samples (e.g., psychiatric epidemiological samples, non–comorbid PTSD samples) may provide some advantages over our sample, they also have limitations. For example, psychiatric epidemiological samples may have much lower representation of people with PTSD. We strongly encourage further research on the identification of factors and symptoms related to PTSD underdiagnosis across a wider variety of samples and study types. Such triangulation of studies across data sources aligns with the approaches of workgroups that informed previous editions of the *DSM* (APA, 1980, 1994, 2013).

Another limitation relates to the fact that, given that the majority of contributing trials in Project Harmony were conducted before the 2013 release of *DSM‐5*, direct assessment of the two “priority” symptoms (i.e., NACM and persistent negative feelings) was limited. Additionally, the RCTs included in this larger meta‐analysis included adults with comorbid PTSD and SUDs rather than individuals with PTSD alone or those with PTSD and other psychiatric or physical comorbidities. Future research is needed to assess whether the priority symptoms identified in this work are replicated among other samples and presentations of PTSD, particularly using an integrative data analysis approach to ensure sufficient statistical power. Next, the use of dichotomized symptom data may limit sensitivity to individual variations in symptom frequency or intensity. However, this approach was necessary to harmonize assessments with incompatible scoring metrics, such as the CAPS (separate frequency and intensity scores) and the CAPS‐5 (integrated frequency and intensity). Standardizing to a binary threshold provided the most robust common denominator for comparisons across different assessment approaches (Bauer & Hussong, 2009).

Lastly, although prior research (Morgan‐López, Killeen et al., 2020) has examined sociodemographic characteristics (e.g., sex, age, veteran status) of underdiagnosed individuals, this study did not analyze the specific impact of the trauma type on class membership. Future research is needed to determine if distinct types of trauma exposure (e.g., combat vs. interpersonal violence), cumulative trauma history (i.e., single incident vs. polyvictimization), or structural and systemic forms of trauma are differentially associated with the subthreshold symptom profiles identified here.

Despite these limitations, our findings emphasize the importance of including individuals with subthreshold PTSD in clinical research, especially RCTs, and testing alternate approaches for classifying PTSD to ensure that individuals experiencing significant distress are accurately identified so they can access appropriate treatment for their presenting problems. Although these findings are initial and require replication, they are an important part of the conversation regarding the clinical utility of diagnoses and how subthreshold conditions are handled in health care settings. If replicated across diverse epidemiological samples, these patterns could support policy recommendations regarding diagnoses and insurance reimbursement to include subthreshold PTSD as a clinically significant condition requiring intervention; this is an important step toward reducing barriers to care for individuals who experience meaningful distress but do not meet *DSM* diagnostic thresholds.

Future research should also include individuals who might not meet the *DSM*‐based criteria but report substantial distress and impairment in intervention trials, especially when focused on comorbid presentations. The inclusion of these individuals is likely to improve the discriminant validity of findings and allow researchers to evaluate whether and how evidence‐based treatments, such as prolonged exposure (Foa et al., 2019) or cognitive processing therapy (Resick et al., 2024), may benefit individuals with distinct PTSD symptom profiles. Additionally, diagnostic and clinical assessments could pivot toward dimensional models that consider variations in symptom severity rather than relying solely on categorical diagnostic thresholds. This could result in enhanced diagnostic precision and allow providers to recommend treatment strategies that better align with their participants’ needs. Implementing these considerations could lead to a more inclusive, accurate, and effective framework for diagnosing and treating individuals who experience traumatic stress and PTSD symptoms.

## AUTHOR NOTE

This work was funded by grants from the National Institute on Drug Abuse (Clinical Trials Network Protocol 0015; 2UG1DA04946; 1R01DA061338; R01DA057308) and the National Institute of Alcohol Abuse and Alcoholism (NIAAA; R01AA025853; T32AA007453; R25AA028464; R00AA029459).

The Consortium on Addiction, Stress, and Trauma (CAST) includes: Steven Batki, Malcolm Battersby, Matthew Boden, Kathleen Brady, Deborah Brief, Christy Capone, Kathleen Chard, Joan Cook, Annette Crisanti, Erica Eaton, Thomas Ehring, Paul Emmelkamp, Edna Foa, Linda Frisman, Jessica Hamblen, Moira Haller, Teresa López‐Castro, Deborah Kaysen, Shannon Kehle‐Forbes, Annett Lotzin, Asa Magnusson, Meghan McDevitt‐Murphy, Mark McGovern, Katherine Mills, Lisa Najavits, David Oslin, Jessica Peirce, Ismene Petrakis, M. Zachary Rosenthal, Lesia Ruglass, Michael Saladin, Claudia Sannibale, Rebecca Schacht, Ingo Schaefer, Jeremiah Schumm, Tracy Simpson, Susan Sonne, Geraldine Tapia, Jessica Tripp, Debora Van Dam, Anka Vujanovic, and Caron Zlotnick.

## OPEN PRACTICES STATEMENT

Due to confidentiality agreements with some of the original studies, a smaller harmonized and deidentified dataset will be made available for sharing on request from the principal investigators of the parent grant: **Denise A. Hien** (denise.hien@rutgers.edu) and/or **Antonio A. Morgan‐López** (amorganLópez@rti.org).

## AUTHOR CONTRIBUTIONS

**Krithika Prakash**: methodology, visualization, writing–original draft, writing–review and editing. **Antonio A. Morgan‐López**: funding acquisition, conceptualization, formal analysis, writing–original draft, writing–review and editing, supervision. **Jordan A. Gette**: writing–original draft, writing–review and editing. **Kelly R. Peck**: writing–original draft, writing–review and editing. **Marilyn L. Piccirillo**: writing–original draft, writing–review and editing. **Lissette M. Saavedra**: conceptualization, writing–review and editing. **Sudie E. Back**: writing–review and editing. **Therese K. Killeen**: writing–review and editing. **Sonya B. Norman**: writing–review and editing. **Stephen G West**: writing–review and editing. **Denise A. Hien**: funding acquisition, conceptualization, writing–review and editing.

## Supporting information



SUPPORTING INFORMATION
