# Supplementary material for: Impaired but now diagnosable: Combining analytic frameworks for incorporating symptom weighting in the classification of full‐threshold and subthreshold posttraumatic stress disorder
Source: J Trauma Stress. 2026 Aug 5;39(5):962–74. doi: 10.1002/jts.70112 (PMC13639219; doi:10.1002/jts.70112)
Supplement: Supplementary file 1 [file JTS-39-962-s001.docx]

Supplemental Material

*Table S1. Fit Indices, Entropy, and Posterior Probabilities of Latent Classes Analysis*

| # of Classes | Akaike Information Criterion (AIC) | Bayesian Information Criterion (BIC) | Entropy | Posterior Probability Range (excluding Class 1) |
| --- | --- | --- | --- | --- |
| 2 | 26615 | 26844 | 1 | 1 |
| 3 | 26462 | 26808 | .989 | .930-.960 |
| 4 | 26387 | 26850 | .989 | .919-.949 |
